# RAVA: Region-Based Average Video Quality Assessment

**DOI:** 10.3390/s21165489

**Published:** 2021-08-15

**Authors:** Xuanyi Wu, Irene Cheng, Zhenkun Zhou, Anup Basu

**Affiliations:** 1 Department of Computing Science, University of Alberta, Edmonton, AB T6G 2R3, Canada; locheng@ualberta.ca (I.C.); basu@ualberta.ca (A.B.); 2 Huawei Fields Laboratory, Hangzhou 310000, China; kfzle@126.com

**Keywords:** video quality assessment, objective score, human visual system (HVS), image quality assessment

## Abstract

Video has become the most popular medium of communication over the past decade, with nearly 90 percent of the bandwidth on the Internet being used for video transmission. Thus, evaluating the quality of an acquired or compressed video has become increasingly important. The goal of video quality assessment (VQA) is to measure the quality of a video clip as perceived by a human observer. Since manually rating every video clip to evaluate quality is infeasible, researchers have attempted to develop various quantitative metrics that estimate the perceptual quality of video. In this paper, we propose a new region-based average video quality assessment (RAVA) technique extending image quality assessment (IQA) metrics. In our experiments, we extend two full-reference (FR) image quality metrics to measure the feasibility of the proposed RAVA technique. Results on three different datasets show that our RAVA method is practical in predicting objective video scores.

## 1. Introduction

Video is widely used in our daily lives. TV shows, computer games, online meetings, all rely on the quality of video. As mentioned in [1], video sensor networks (VSNs) are communication infrastructures that involve video coding, transmission, and display/storage. Via VSNs, the dense visual information is captured and transmitted to applications on different devices for users to view. The size of video clips is many times larger than that of images and texts. To reduce bandwidth usage and save storage space, video coding is widely used. Recent technologies such as that in [2] have attempted to transmit only the difference in data between the past and current images, while the difference data are calculated by a MPEG-4 Visual video encoder. Some technologies are integrated into the encoders for video compression. For example, a denoising algorithm is combined with a high-efficiency video coding (HEVC) encoder to improve the compression efficiency in [3]. More importantly, if the quality of the network is poor, the perceptual quality continues to deteriorate as fewer packets are received [4]. This is another problem we face during transmission. Before displaying to users, the decoder restores the data to a video. However, the quality of the video is often degraded after such a long process. The question is, “How can we measure quality?”.

In the real world, objects are three-dimensional (3D). To measure the perceptual quality, many aspects need to be considered. Resolutions of texture and mesh are combined to measure 3D perceptual quality in [5]; optimized linear combinations of accurate geometry and texture quality measurements are used in [6]; and a multi-attribute computational model optimized by machine learning is used in [7]. For videos, all 3D objects are projected onto a 2D plane. Thus, estimating the quality for videos is simpler than quality estimation for 3D objects. Quality assessment can be performed considering two types of scores—subjective and objective. Users need to manually rate videos to allow the computation of precise subjective scores; however, this process can be very time-consuming. Thus, researchers have tried to develop objective scores that can estimate subjective scores automatically. Many aspects affect objective scores, such as contrast, frequency, pattern, and color perception. Thus, some metrics are developed to analyze specific features. Objective metrics can be further divided into three categories: full-reference (FR) methods; reduced-reference (RR) methods; and no-reference (NR) methods.

There are many image quality assessment (IQA) methods. For instance, SSIM [8] and PSNR [9] are FR IQA methods, [10,11] and NIMA [12] are NR IQA methods. Since videos are composed of image frames, we consider extending IQA methods to assess video quality. Some VQA methods are extended from IQA methods, including PSNR [9], the extension of PSNR based on HVS (PSNR-HVS) [13], PSNR based on between-coefficient contrast masking (PSNR-HVS-M) [14]), structural similarity image metrics (SSIM) [8], and the extension of SSIM to video structural similarity (VSSIM) [15]). However, existing full-reference VQA methods do not have high correlation with human perception while the video content varies considerably. In addition, some methods simply average the IQA scores for all the frames. By doing this, they only consider spatial features such as color and illumination, but neglect the temporal features. Our goal is to combine motion features and 2D spatial features together. In our work, we extend two IQA methods, namely SSIM [8] and PSNR [9], to obtain the RAVA scores. The reason we choose these IQA metrics are outlined below. SSIM and PSNR are the most widely used IQAs. We want to apply them to video quality evaluation and compare their performance with existing VQA methods, especially with the VQA methods extended by them. We divide each frame into foreground and background regions, calculate the IQA scores for those regions, and then assign them different weights based on the motion features. We also notice that if we linearly combine foreground and background scores as the VQA scores, the range and mean value differ considerably for videos with different content. To generalize the VQA scores for videos with various types of content, we introduced a self-supervised video content distinction network. Finally, the foreground, background, and content distinction features are passed to a support vector regression (SVR) [16] to obtain the final VQA score.

For evaluation, we used the LIVE Mobile VQA database [17], the MCL-V database [18], and the Netflix Public Dataset [19]. The video categories in these datasets are quite varied. These datasets are widely used to analyze video quality. The LIVE Mobile VQA database [17] and the Netflix Public Dataset [19] provide the subjective differential mean opinion score (DMOS), while the MCL-V database [18] provides the mean opinion score (MOS). As mentioned in [20], MOS is a typical subjective quality of experience (QoE) assessment score; for instance, users rate quality from 1 (bad quality) to 5 (excellent quality). It is considered the most accurate way to measure QoE since actual users were involved in developing the metric. DMOS is calculated as first getting the difference scores from the raw quality scores, and then converting the scores into Z-scores with outliers removed [21]—here, lower is better. To evaluate the results, we analyzed Pearson’s (linear) correlation coefficient (PCC) [22] and Spearman’s rank correlation coefficient (SCC) [23] of the RAVA scores and the subjective scores.

The contributions of our paper are: (1) proposing a region-based VQA method that estimates video quality by extracting and processing the information of background regions and moving objects in the foreground regions; (2) integrating a self-supervised video content distinction network to generalize the VQA scores for videos with different content; (3) extending two full-reference IQA metrics to VQA metrics in the experiments, which shows the possibility of applying the RAVA technique to other FR IQA methods.

## 2. Related Work

Objective video quality assessment techniques can be categorized into three types: full reference (FR) methods, reduced reference (RR) methods and no reference (NR) methods. FR methods utilize the entire original video to determine the quality score. Nevertheless, their performance is relatively poor in terms of accuracy. Thus, perceptual factors in the human visual system (HVS) need to be incorporated to develop reliable video quality assessment techniques [24]. Some FR VQA methods are as follows.

Netflix proposed video multi-method assessment fusion (VMAF) [19] in 2016. VMAF calculates the visual information fidelity (VIF) [25], detail loss metric (DLM) [26], and a motion feature, which is defined as the average absolute pixel difference for the luminance component between adjacent frames. A support vector regressor (SVR) is subsequently used to fuse these elementary metrics together. In 2018, Netflix posted another blog saying that they added AVX optimization and frame-level multi-threading, which accelerates its execution three times and improves its prediction accuracy [27]. Liu et al. [28] proposed a new VQA metric using space–time slice mappings. They first use spatial temporal slices (STS) [29] to obtain some STS maps. Then, on each of the reference-distorted STS map pairs, they calculated the IQA scores via a full-reference IQA algorithm. Finally, they apply feature pooling on the IQA scores on those maps to obtain the final score. Aabed et al. [30] proposed power spectral density (PSD) [30]. It is a perceptual video quality assessment (PVQA) metric that analyzes the power spectral density of a group of pictures. The authors built 2D time-aggregated PSD (or tempospatial PSD) planes for several sets of frames for both the original and distorted videos to capture spatio-temporal changes in the pixel domain. Following this, they built a local cross-correlation map. The perceptual quality score is the average of the values in the correlation map, with a higher value implying better quality.

RR methods extract some outstanding features from both the original and acquired videos, compare these features and obtain the objective score. For example, the Institute for Telecommunication Science (ITS) proposed the video quality metric (VQM) [31]. It was adopted as the standard by the American National Standards Institute (ANSI) and the International Telecommunication Union (ITU) [31]. VQM is defined in (1):

(1) $$\begin{array}{cc} VQM= & -0.2097*si_{-}loss+0.5969*hv_{-}loss\\ \; & +0.2483*hv_{-}gain+0.0192*chroma_{-}spread\\ \; & -0.3416*si_{-}gain+0.0431*ct_{-}ati_{-}gain\\ \; & +0.0076*chroma_{-}extreme \end{array}$$

Here, *h* and *v* represent the horizontal and vertical axes, respectively; si_loss detects the loss of or decrease in spatial information; si_gain detects edge sharpening or enhancement; hv_loss captures the shift of edges from vertical and horizontal orientations to a diagonal orientation; hv_gain finds the shift of edges from diagonal to horizontal; chroma_spread finds changes in the spread of the distribution of 2D color samples; chroma_extreme measures serious localized color impairments; and ct_ati_gain is the product of a contrast feature [32].

NR methods access the quality of a new video without referring to the original video. Li et al. [33] proposed VSFA (quality assessment of in-the-wild videos). It integrates two eminent effects of the human visual system: content-dependency and temporal-memory effects. Content-dependency effects are obtained by extracting features from a pre-trained image classification neural network on ImageNet; temporal-memory effects are integrated by adding a gated recurrent unit and a subjectively inspired temporal pooling layer to the neural network. The method does not refer to the original video when predicting the video quality. Zadtootaghaj et al. [34] proposed an NR VQA method DEMI. DEMI first uses the scores predicted by the pre-trained VMAF model [19] for training. Then, it is fine-tuned on a small image quality dataset. Finally, the authors apply random forest for feature pooling.

The problem for existing FR and RR methods is that they do not working well if the content of videos in a dataset varies a lot. The correlation values of many existing FR and RR VQA scores with human perception are low. For the NR methods, the predicted quality tends to be more affected by the content than the distortions. NR methods are often trained and tested with in-the-wild video datasets. The videos there are collected from real-world video sequences. The content does vary significantly, but we are not sure how much distortion is involved.

Since we want to extend some image quality assessment metrics to video, we will introduce the two full-reference IQA metrics we used below.

- PSNR
  Peak signal-to-noise ratio (PSNR) [9] is the ratio between the maximum possible power of a signal and the power of the corrupting noise that affects the fidelity of its representation. A higher value of PSNR is better:
  (2) $$PSNR=20\log_{10}\left(\frac{MAX_{f}}{\sqrt{MSE}}\right)$$
  where $MAX_{f}$ is the highest value in the two input variables (it is normally 255 for RGB images) and MSE is the mean squared error of the two inputs.
- SSIM
  SSIM [8] is a perception-based model that considers image degradation as perceived change in structural information, while also incorporating important perceptual phenomena, including both luminance and contrast masking. Structural information is the idea of pixels having strong inter-dependencies, especially when they are spatially close. For SSIM, a higher value is better:
  (3) $$\mathrm{SSIM}\left(x,y\right)=\frac{\left(2\mu_{x}\mu_{y}+c_{1}\right)\left(2\sigma_{xy}+c_{2}\right)}{\left(\mu_{x}^{2}+\mu_{y}^{2}+c_{1}\right)\left(\sigma_{x}^{2}+\sigma_{y}^{2}+c_{2}\right)}$$
  where $\mu_{x}$ is the average of *x*, $\mu_{y}$ is the average of *y*, $\sigma_{x}^{2}$ is the variance of *x*, $\sigma_{y}^{2}$ is the variance of *y*, $\sigma_{xy}$ is the covariance of *x* and *y*, $c_{1}$ and $c_{2}$ are two variables to stabilize the division with weak denominator, with $c_{1}={\left(k_{1}L\right)}^{2}$, $c_{2}={\left(k_{2}L\right)}^{2}$. *L* is the dynamic range of the pixel-values (typically $L=2^{\#bits\;per\;pixel}-1$). $k_{1}=0.01$, $k_{2}=0.03$ by default.

## 3. Proposed Method

**Figure 1:**
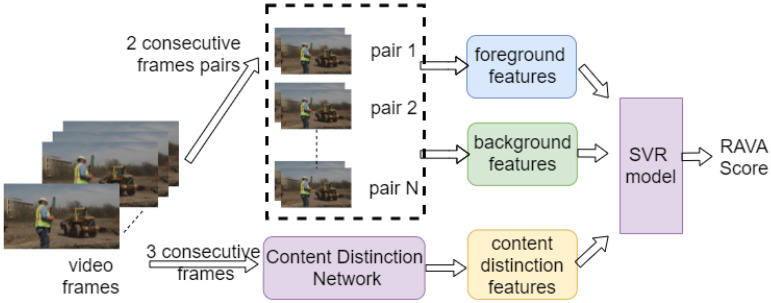
Main procedure.

Figure 1 illustrates the main procedure of the proposed method. The key idea is to compute a region-based weighted average, combining spatial and temporal features while integrating content distinction features. At the beginning, each frame is divided into several regions. Motion features are extracted by optical flow [35]. Later, they can be used to define the weights for each region. Following this, larger weights can be assigned to regions with larger motion change and smaller weights can be assigned to regions with smaller motion change. Furthermore, videos with similar content tend to have similar VQA scores. How to assess the quality of videos with different contents has become a problem. Motivated by this, we add a content distinction neural network to generalize the VQA scores for videos with varying content.

### 3.1. Foreground Features

The first step is to define and find the foreground regions. Humans tend to pay more attention to the foreground objects than the background, so we want to define the regions based on this observation. In our implementation, for every consecutive pair of frames, $I_{i}$ and $I_{i+1}$, we first locate the objects in frame $I_{i}$ with bounding boxes. Then, the boxes are used to approximate those objects’ positions in the next frame $I_{i+1}$ and also to define the regions used to calculate the optical flows. Each region in $I_{i}$ can be represented as

(4) $$R_{i,k}=\left\{\begin{array}{cc} x_{L}\leq x\leq x_{L}+w & \\ y_{L}\leq y\leq y_{L}+h \end{array}\right.$$

where *k* is the kth region in frame $I_{i}$, $\left(x_{L},y_{L}\right)$ is the top left corner of the bounding box, *w* is the width and *h* is the height.

**Figure 2:**
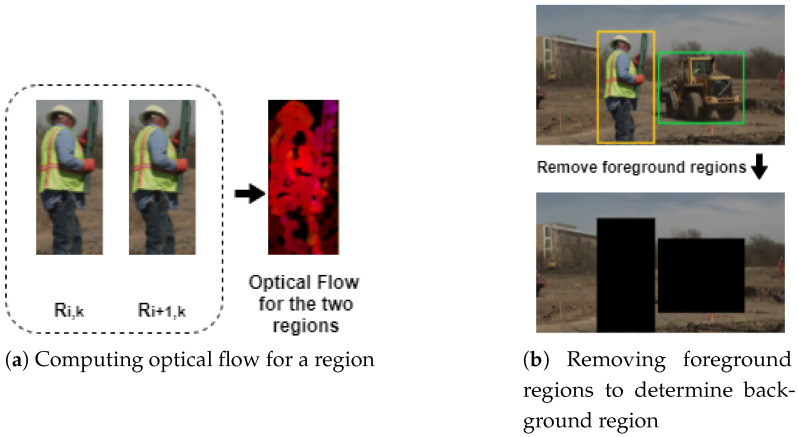
(**a**) Shows how to get the motion feature for the foreground region; and (**b**) defines the background region.

After finding the regions, we need to determine their weights. Since we are dealing with video quality, we cannot only consider the spatial features. To address the relationship between frames, we use optical flow to calculate the weights, as shown in Figure 2a. Optical flow shows the pattern of apparent motion changes of objects, surfaces, and edges caused by the relative motion between an observer and the scene [36,37]. In addition, it gives us information about the rate of the change of the observer. The rate of change is an important factor in the votes of subjects. If this rate between frames is large, it may cause blurring and affect the viewing experience. Thus, we believe that using optical flow to assign different weights for various regions can reflect the user’s attention in each area to some extent. Flow is represented in polar coordinates, using magnitude and angle. The optical flow for the $k^{th}$ region in frame $I_{i}$ can be represented as

(5) $$mag_{i,k},\alpha_{i,k}=f_{R_{i,k}\to R_{i+1,k}}$$

where $R_{i+1,k}$ is the same region in the next frame $I_{i+1}$. The average magnitude for this region is:

(6) $$avg_{-}opt_{i,k}=\frac{\sum_{j=1}^{K}mag_{i,k,j}}{K}$$

where *j* is the iterator to go through the values in $mag_{i,k}$ and *K* is the number of pixels in it.

We do not consider the foreground regions and weights in the last frame, since optical flow calculation needs two frames. After we obtain the average magnitudes for all the foreground regions, the weight assigned for region $R_{i,k}$ is:

(7) $$w_{i,k}=\frac{avg_{-}opt_{i,k}}{\sum_{m=1}^{M-1}\sum_{n=1}^{N}avs.g_{-}opt_{m,n}}$$

where *M* is the number of frames and *N* is the number of regions detected in frame *m*. The foreground feature can be calculated as a weighted average of the foreground region IQAs:

(8) $$\begin{array}{cc} FG\;feature= & \frac{\sum_{m=1}^{M-1}\sum_{n=1}^{N}w_{m,n}\cdot IQA\left(R_{m,n}\right)}{\sum_{m=1}^{M-1}\sum_{n=1}^{N}1} \end{array}$$

### 3.2. Background Features

We cannot ignore the background regions, especially for those videos with small or no foreground objects. To extract the background only, we mask out the foreground regions with zeros, as shown in Figure 2b. The background region for frame $I_{i}$, which we call $BG_{i}$, is defined by the following equations:

(9) $$BG_{i}=I_{i}⊖R_{i,k}\;for\;k\in \left[1,number\;of\;regions\right]$$

where ⊖ is defined as region-based pixelwise manipulation, with the restriction: pixel at location (x,y) in image $I_{i},$ namely $I_{i,x,y}=max\left(0,I_{i,x,y}-R_{i,k,x,y}\right)$.

The background feature is the simple average IQA for background regions:

(10) $$\begin{array}{cc} BG\;feature= & \frac{\sum_{m=1}^{M}IQA\left(BG_{i}\right)}{M} \end{array}$$

Algorithm 1 shows the pseudocode for getting the foreground and background features.
**Algorithm 1:** Proposed RAVA algorithm.RAVA(I,M):    *I* represents list of all the frames of a video, and *M* is the number of frames.  1:        2:i←1  3:**for all**i←1toM−1**do**  4: $Bboxes_{i}\leftarrow locate\;objects\;in\;frame\;I_{i}$  5: $BG_{i}\leftarrow I_{i}$  6: **for all** $k\leftarrow 1\;to\;len(Bboxes_{i})$ **do**  7:  $R_{i,k}\leftarrow Bboxes_{i}\left[k\right]$  8:  $BG_{i}\leftarrow I_{i}⊖R_{i,k}$                {Mask out foreground regions}  9:  $mag_{i,k},\;\alpha_{i,k}\leftarrow Optical\;flow\left(\;R_{i,k}\to R_{i+1,k}\right)$10:  $avg_{-}opt_{i,k}\leftarrow \frac{\sum_{j=1}^{K}mag_{i,k,j}}{K}$11:                    {K is the number of pixels in this region $R_{i,k}$}12: **end for**13:**end for**14:**for all**i←1toM−1**do**15: **for all** k←1toN **do**16:                      {N is the number of regions in the frame}17:  $w_{i,k}=\frac{avg_{-}opt_{i,k}}{\sum_{m=1}^{M-1}\sum_{n=1}^{N}avs.g_{-}opt_{m,n}}$18:      19: **end for**20:**end for**21:$FG\;feature=\frac{\sum_{m=1}^{M-1}\sum_{n=1}^{N}w_{m,n}\cdot IQA\left(R_{m,n}\right)}{\sum_{m=1}^{M-1}\sum_{n=1}^{N}1}$22:      23:$BG\;feature=\frac{\sum_{m=1}^{M}IQA\left(BG_{i}\right)}{M}$24:                             {IQA can be PSNR or SSIM}

### 3.3. Content Distinction Features and Self-Supervised Learning

#### 3.3.1. Statistical Analysis

**Figure 3:**
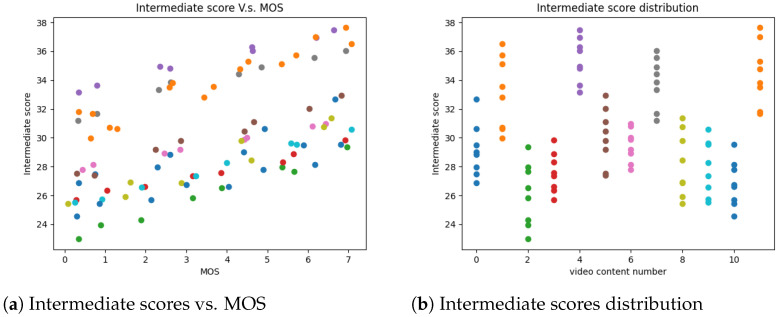
Statistical analysis.

We first attempted to linearly combine the foreground and background features as the intermediate RAVA score. However, the result was not good. Figure 3a shows the plot for the intermediate RAVA${}_{PSNR}$ scores vs. MOS on the MCL-V [18] database, where dots with the same colors represent videos distorted from the same raw HD video. We can see that videos with the same content fit a line, however, videos with different contents are scattered. Thus, we performed a statistical analysis to determine the causes. Figure 3b shows the intermediate RAVA${}_{PSNR}$ scores for the distorted videos in the MCL-V database. Scores for videos with the same content are in the same bin. There are 12 bins, since there are 12 raw videos. We can notice that even under the same distortions, the distribution for the intermediate scores are different. They have distinct means and ranges. Motivated by this finding, we chose the mean and range to be the content distinction features. There are also some existing works such as MaD-DLS [38], a full-reference image quality assessment method, which analyzes the mean and range (deviation) when designing the metric.

#### 3.3.2. Self-Supervised Learning for Content Distinction Features

**Figure 4:**
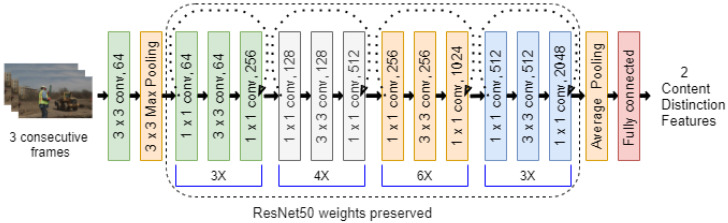
Self-supervised content distinction network.

To predict the aforementioned features, we applied transfer learning on ResNet50 [39]. We modified the architecture somewhat. Since we were dealing with the content for videos, the number of layers for the inputs was set to nine instead of three. We used three consecutive frames in a video to predict its content distinction features. We also modified the final fully connected layer to keep only two values, representing the mean and range. The middle layers are the same as ResNet50. Their weights are loaded from the pre-trained ResNet50 on ImageNet, and kept unchanged. Only the weights for the input layer, the average pooling layer, and the final fully connected layer are tuned by learning on the video datasets, as shown in Figure 4. By doing this, the content distinction network can learn faster.

This is a self-supervised learning process, meaning that we do not train the network with the ground truth (DMOS or MOS). Instead, as mentioned above, we use the means and ranges of the intermediate scores to be the content distinction features. The implementation details are discussed in Section 4.2.2.

### 3.4. Feature Pooling with the SVR Model

In recent years, machine-learning algorithms became popular for feature pooling, as they can consider the strength of those features and assign different weights. For instance, both the full-reference VQA method VMAF [19,27] and the no-reference VQA method TLVQM [40] use SVR for feature pooling to develop a final metric. In our work, the foreground, background, and content distinction features are passed to a support vector regression (SVR) with an RBF kernel, which allows non-linear mapping.

## 4. Experimental Results

### 4.1. Description of Datasets

**Figure 5:**
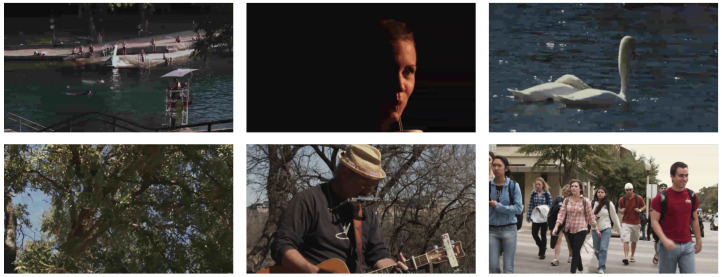
Snapshots from the LIVE database.

The proposed RAVA methods were evaluated on two existing datasets: the LIVE Mobile Video Quality Assessment (VQA) Database [17] and the MCL-V database [18]. The LIVE Mobile Video Quality Assessment (VQA) Database consists of 10 RAW HD reference videos and 200 distorted videos (four compression, four wireless packet-loss, four frame-freezes, three rate-adapted and five temporal dynamics per reference). Each video has a resolution of 1280 * 720 at a frame rate of 30 fps and a duration of 15 s [17]. The study involved over 50 subjects, resulting in 5300 summary subjective scores and time-sampled subjective traces of video quality [17]. The dataset involves two different evaluation approaches to obtain DMOS: mobile and tablet. We analyzed the results for both of these approaches in our experiments. Figure 5 shows some snapshots from this dataset.

**Figure 6:**
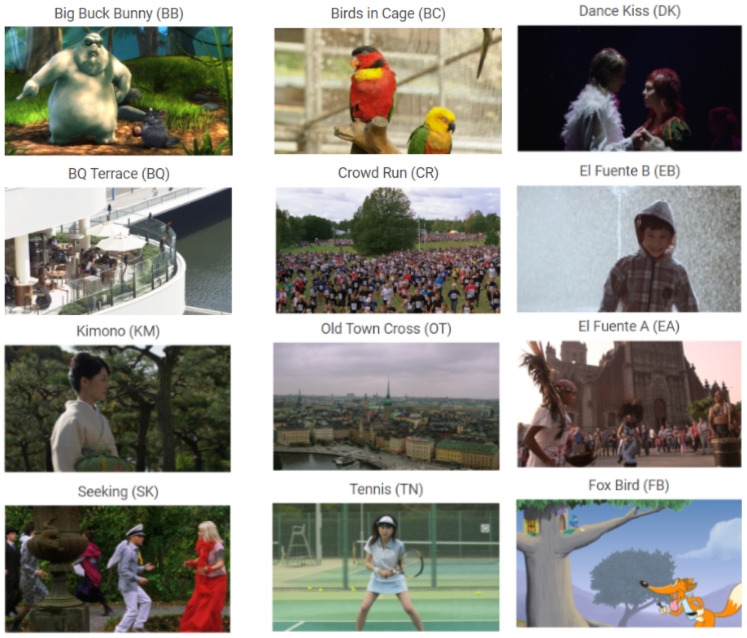
Snapshots from the MCL-V database.

The MCL-V database contains 12 uncompressed HD (1920 * 1080) source video clips, as shown in Figure 6. Its resolution is higher than the previous dataset. In addition, this dataset captures two typical video distortion types—compression and image size scaling. For each distortion type, four distortion levels are adopted, resulting in 96 distorted video clips in total. Furthermore, its contents are quite varied. There are not only real-life video clips, but also some cartoons and animations. It also provides the mean opinion scores (MOSs) along with the videos.

The Netflix Public Dataset is a full-reference video quality assessment dataset published by Netflix together with their work [19,27]. It consists of nine source video clips of resolution 1920×1080 with frame rates ranging from 24 to 30 fps. The source clips are encoded in multiple resolution–bitrate pairs. The bitrates go from 375 to 5800 kbps while the resolution goes from 288 to 1080 p. They also provide the differential mean opinion scores (DMOSs) for the 70 distorted videos. Note that lower DMOS values are normally better, as mentioned in [21]. However, for the DMOSs provided in the Netflix Public Dataset, higher values are better. Their range varies from 10 (impairments are annoying) to 100 (impairments are imperceptible) [19]. We used this dataset for cross-library evaluation.

### 4.2. Implementation Details

#### 4.2.1. Packages

There do exist some advanced object detection and tracking methods such as AdaMM [41] and content-aware focal plane selection [42]. They can handle complex situations such as occlusion. However, in our work, an object does not affect the viewing experience if it is not visible. Moreover, the position of an object does not greatly differ for two consecutive frames, so we simply used YOLOv3 [43] to locate an object with a bounding box and used that location to approximate its position in the next frame. The bounding boxes’ widths and heights are offsets from the anchor boxes’ centroids. YOLOv3 uses anchor boxes of nine different sizes. The smallest anchor box is of size 10×13 so that it can track small objects. If the object is even smaller than this size, then it is viewed as a background, as discussed in Section 3.2. We tried two implementations to calculate the optical flow: dense optical flow provided by OpenCV [44] and FlowNet2 [45]. Their performances are very similar. In the following paragraphs, we showed the result with the first implementation.

#### 4.2.2. Training on the Content Distinction Network

**Figure 7:**
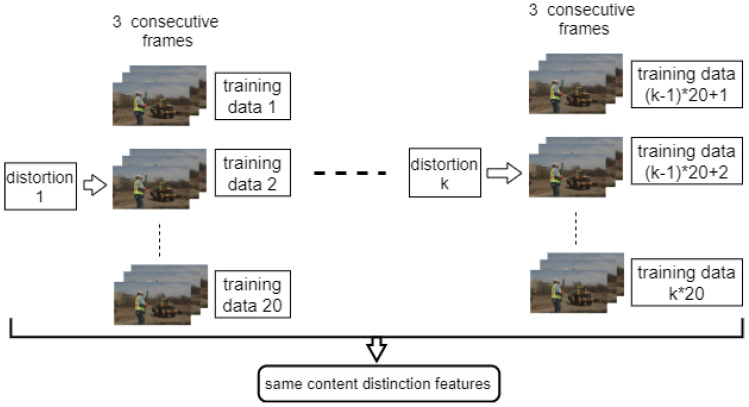
Preparing training data for videos with the same content (distorted from the same RAW video).

**Figure 8:**
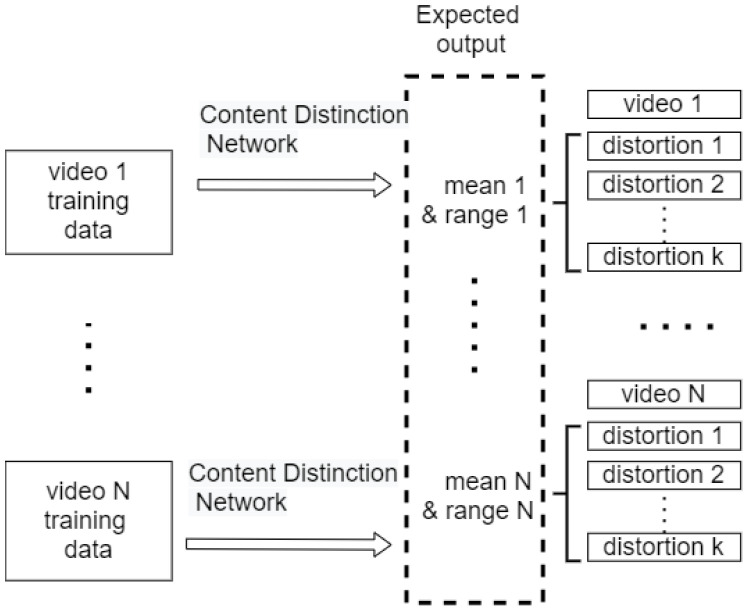
Content distinction network learning procedure.

The features are called “content distinction”; thus, regardless of the distortion type, the video with the same content should obtain the same features. Figure 7 and Figure 8 show how we prepare the training data. To obtain the training data for Video 1, we randomly generate 20 groups of frames from each video distorted from it. Each group contains three consecutive frames. For example, if we have eight videos distorted from Video 1, we will have 8×20=160 groups. To save some training time, each frame is resized to 240×240. All the groups will have the same content distinction features, so we expected that they would have the same output from the network. The features we used for training are the mean and range of all the intermediate scores for a video. We repeated the process for all the videos with different content.

We used the Adam optimizer with a fixed learning rate of 0.0005 for 20 epochs to train the models. We perform cross-dataset prediction when we generated the content distinction features. This means that we trained the content distinction network on one dataset and predicted features on another dataset. In this case, the contents that we learned and tested were quite different. Our experimental results show that this is a promising direction.

#### 4.2.3. SVR Model Parameter Tuning

The SVR models on the LIVE mobile database and the MCL-V database are separately tuned since one dataset provides MOS and the other provides DMOS. For each dataset, we perform a random train–test split: 80% of the data train the SVR model, and the remaining data are used for testing. We calculated the average PCC and SCC over 100 runs. Each run has a different random seed. This can reduce the effect of some special cases and show off the overall performance. γ and *C* are the two parameters to be tuned. γ is the inverse of the standard deviation of the Gaussian function. *C* is used to control the regularization term. There are two common techniques for parameter tuning, namely grid search and random search. Both can help tune the hyper-parameters by trying different values and picking the one with the best performance. However, since random search randomly tries potential values, it can miss the best values. Thus, we used grid search.

### 4.3. Evaluation Criteria

We used Pearson’s (linear) correlation coefficient (PCC) [22] and Spearman’s rank correlation coefficient (SCC) [23] to see the correlation of the two RAVA scores with DMOS or MOS. PCC and SCC are the most popular methods for measuring the dependence of two variables *X* and *Y*. PCC evaluates the linear relationship while SCC evaluates the monotonic relationship. Mathematically, PCC can be written as

(11) $$\mathrm{PCC}\left(X,Y\right)=\frac{\mathrm{cov}(X,Y)}{\sigma_{X}\sigma_{Y}}=\frac{E[\left(X-\bar{X}\right)\left(Y-\bar{Y}\right)]}{\sigma_{X}\sigma_{Y}}$$

where $\bar{X}$ and $\bar{Y}$ are the average values of *X* and *Y*, respectively; $\sigma_{X}$ and $\sigma_{Y}$ are the standard deviations.

For SCC, given two samples of size *n* for both *X* and *Y*, $R_{X_{i}}$ denotes the rank of $X_{i}$ in the ascending sorted *X* sample. Similarly, $R_{Y_{i}}$ denotes the rank of $Y_{i}$. When several observations have the same rank, an average rank will be assigned to them. Mathematically, SCC can be written as

(12) $$SCC=1-\frac{6\sum_{i=1}^{n}d_{i}^{2}}{n\left(n^{2}-1\right)}$$

where $d_{i}=R_{X_{i}}-R_{Y_{i}}.$

RAVA scores and MOS or DMOS may increase by very different factors. In this case, the PCC value will be affected. However, since SCC is calculated based on the ranks, the value for SCC will remain the same even if they change at different rates. Taking this into consideration, we also reported the SCC scores. In addition, the correlation coefficients are in range of −1–1 since the RAVA scores and the ground truth scores may go in different directions. We only want to measure how strongly the two variables are correlated. Thus, we will only compare the absolute value of PCC and SCC values. If the absolute value is large, then their correlation is high; otherwise, they are less likely to be correlated. The new metric is still considered to be valuable if the negative correlation is strong.

### 4.4. Experimental Results

Table 1 shows the values we use for the two parameters γ and *C* for the LIVE mobile database and the MCL-V database.

#### 4.4.1. The LIVE Mobile Database (Mobile DMOS)

**Figure 9:**
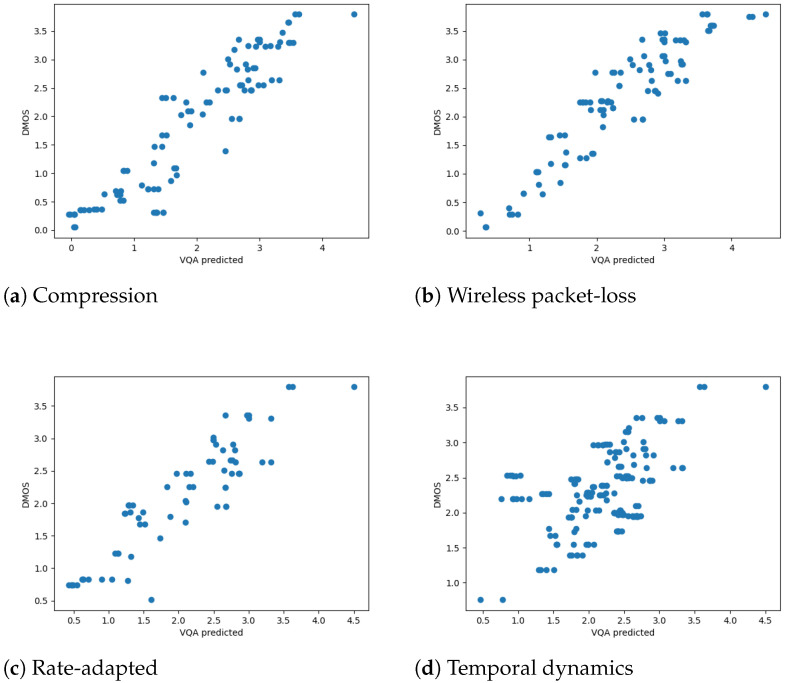
RAVA${}_{SSIM}$ vs. DMOS for different distortion types (mobile).

**Figure 10:**
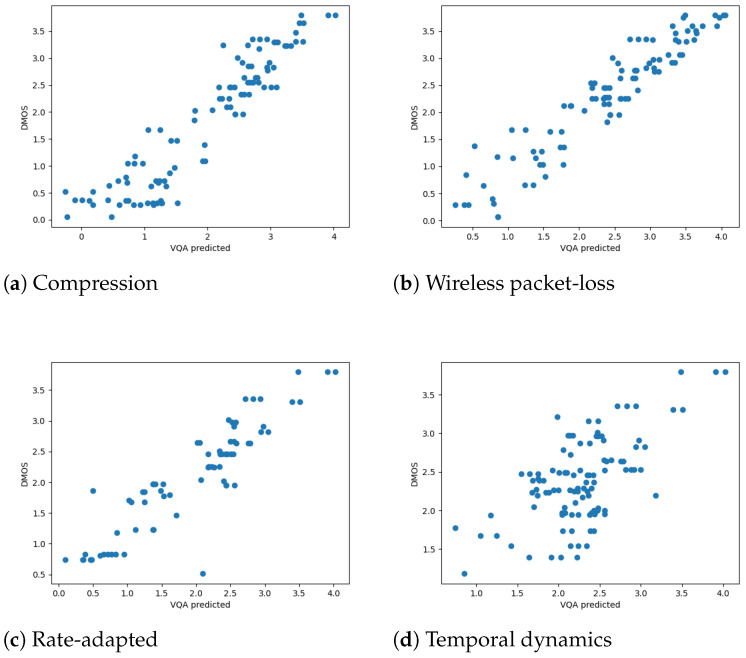
RAVA${}_{PSNR}$ vs. DMOS for different distortion types. (mobile).

**Figure 11:**
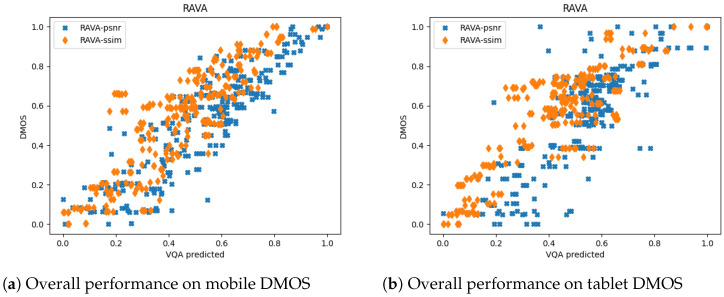
The overall performance of the RAVA models on the Live Mobile Database (mobile and tablet).

We first analyzed the SCC and PCC on the mobile DMOS for the LIVE Mobile Video Quality Assessment (VQA) Database. To better visualize the results for different distortion types, we drew the scatter plots of the RAVA scores vs. DMOS in Figure 9 and Figure 10. The plots aggregate the test results for 10 runs. As mentioned in [17], how humans rate videos with freeze-frame distortions is still unclear, as we only draw plots for the other four distortion types. The overall performance of the two RAVA methods are drawn in Figure 11a for comparison. The two methods have different ranges. Thus, to visualize using the same scale, we normalized the DMOS and RAVA scores before drawing the plot.

From all the plots, we can see that the performance of the two proposed FR VQA methods RAVA${}_{SSIM}$ and RAVA${}_{PSNR}$ are very similar. They performed well on videos distorted with compression, wireless packet-loss, and rate adaptation. Furthermore, we compared our PCC and SCC results with eight commonly used video quality assessment methods: PSNR [9]; VQM [32]; MOVIE [46]; MS-SSIM [47]; SS-SSIM [8]; VIF [25]; VSNR [48]; and NQM [49]. The quantitative comparisons are shown in Table 2 and Table 3. The results for the two RAVA methods are averaged over 100 runs. A bold value in a column represents the highest value in that column. Note that Co means compression; wl means wireless channel packet loss; Ra means rate adaptation; and Td means temporal dynamics.

As shown in the tables, RAVA${}_{SSIM}$ outperformed all the listed VQA methods for both SCC and PCC. Moreover, it achieved the best performance in all the distortion types except for temporal dynamics. Compared to the existing SS-SSIM method, the overall correlation was increased by 0.169 and 0.195 for SCC and PCC, respectively. Furthermore, our RAVA${}_{SSIM}$ was improved by 0.076 (SCC) and 0.151 (PCC) compared to MS-SSIM. RAVA${}_{PSNR}$ also performed well, though its overall performance ranked second. The correlation was increased by 0.134 (SCC) and 0.152 (PCC) compared to the existing PSNR method.

Note that all the existing VQA methods do not perform well for the temporal dynamics distortion. The best performance is 0.386 (VQM) in SCC and 0.427 (VSNR) in PCC. The two proposed RAVA methods obtained higher scores compared to all the existing methods in videos distorted by this type, as all the SCC values were above 0.5 and all the PCC values were above 0.6.

#### 4.4.2. The LIVE Mobile Database (Tablet DMOS)

Similar to the above experiments, we obtained the SCC and PCC for the tablet DMOS considering the average of 100 runs. The results are shown in Table 4 and Table 5. There is also a plot comparing the overall performance of the two RAVA methods for 10 runs, as shown in Figure 11b.

When predicting the DMOS on tablet devices, most existing methods do not perform well, especially for videos distorted by temporal dynamics. However, the performance of the RAVA methods do not degrade very much. RAVA${}_{SSIM}$ performs well on videos distorted by compression and wireless packet-loss. RAVA${}_{PSNR}$ outperforms all the other methods in videos distorted by temporal dynamics. If we look at the overall performance, the two RAVA methods have the top two scores in both SCC and PCC.

#### 4.4.3. The MCL-V Database

The experimental results on the MCL-V database over 100 runs are shown in Table 6. We compared our result with the following VQA methods: PSNR [9]; SS-SSIM [8]; MS-SSIM [47]; VIF [25]; VADM [50]; and FSIM [51].

As suggested in [18] and [52], we applied the following non-linear regression on the VQA scores before calculating the PCC and SCC scores for all the VQA metrics when evaluating on this dataset:

(13) $$y=\beta_{1}\cdot \left(0.5-\frac{1}{1+e^{\beta_{2}\left(x-\beta_{3}\right)}}\right)+\beta_{4}\cdot x+\beta_{5}$$

$\beta_{1}$–$\beta_{5}$ are the five fitting parameters and *x* is the objective VQA score.

In Table 6, RAVA${}_{PSNR}$ has the best performance. It has the best overall performance for videos with distortion scaling in both PCC and SCC. RAVA${}_{SSIM}$ also performs well. It is not as good as RAVA${}_{PSNR}$ in terms of dealing with videos distorted by scaling, but exceeds it in predicting videos with compression. Both of our methods have better performance than existing PSNR, SS-SSIM, and MS-SSIM methods. RAVA${}_{PSNR}$ outperforms PSNR by 0.293 in PCC and 0.331 in SCC. RAVA${}_{SSIM}$ improves SS-SSIM by 0.099 in PCC and 0.096 in SCC. In addition, it is better than MS-SSIM by 0.128 in PCC and 0.121 in SCC.

The two RAVA methods perform better in the LIVE Mobile database than in the MCL-V database. This is due to the limitations of the training data for MCL-V’s content distinction network. We trained the model with the LIVE Mobile database, but that dataset only contains real-life video clips. Thus, the trained model is not good at predicting the cartoons and animations which are in the MCL-V database.

#### 4.4.4. Cross-Library Experiment on the Netflix Public Dataset

**Figure 12:**
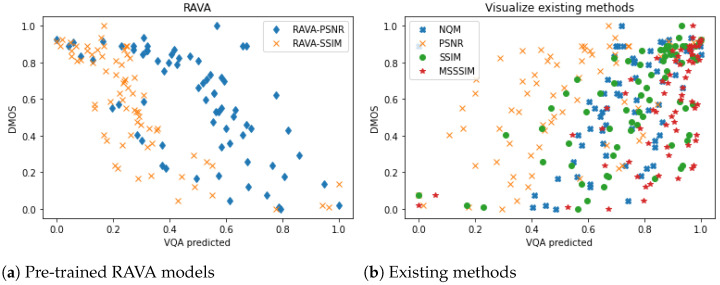
The performance of the pre-trained RAVA models and the existing methods on the Netflix Public Dataset.

We also conducted cross-library validation on the Netflix Public Dataset [19] using the content distinction network and SVR model directly pre-trained on the LIVE Mobile Video Quality Assessment (VQA) database [17]. Figure 12 shows the comparison of the performance of the two pre-trained RAVA models and some existing methods, namely PSNR [9], SS-SSIM [8], MS-SSIM [47], and NQM [49]. Clearly, the dots for the existing methods are more discrete while the dots for the two RAVA methods are more concentrated. Note, as discussed in Section 4.1, that the criteria for collecting the DMOS on the Netflix Public Dataset [19] are different from the criteria for collecting DMOS on the LIVE Mobile Video Quality Assessment (VQA) database [17]. Thus, in our pre-trained model, a lower score is better while for ground truth, higher is better. In Figure 12a, the predicted RAVA scores and the ground truth DMOS are negatively correlated, but we can still see a strong correlation. This can be reaffirmed in Table 7, since the RAVA${}_{PSNR}$ obtains a higher PCC and SCC than PSNR, while RAVA${}_{SSIM}$ performs much better than SS-SSIM and MS-SSIM.

## 5. Conclusions

We introduced a new video quality evaluation approach that integrated various image quality assessment methods, namely region-based detection, temporal weights from optical flow, and content distinction features. Our RAVA technique was applied to extend two full-reference IQA metrics. We first separated foreground and background regions for all the video frames. Then, we integrated the motion features into the weights while designing the VQA metrics. The region weights were defined as the percentage of the average magnitudes of the optical flows for those regions out of all the regions. The foreground feature was the weighted average of the foreground IQA scores, and the background feature was the simple average of background IQA scores. Furthermore, a content distinction network was added to generalize the RAVA scores for videos with various types of content. All the features were passed to an SVR model to predict the final VQA score. We tested on two different datasets to validate the RAVA technique. The LIVE Mobile VQA database and the MCL-V database are widely used VQA datasets, so we used them to compare the performance of the RAVA methods with existing methods. By analyzing the correlation of the RAVA scores and the DMOS (or MOS) provided by the datasets, we noticed that RAVA${}_{PSNR}$ and RAVA${}_{SSIM}$ performed very well. Furthermore, the results produced by RAVA${}_{PSNR}$ were better than those of the PSNR of existing video quality assessment methods. RAVA${}_{SSIM}$ also performed better than SS-SSIM and MS-SSIM.

In summary, we believe that the RAVA approach has practical significance. It can extend IQA methods to VQA methods, and we expect it to be widely applicable for video quality assessment in the future.

## Figures and Tables

**Table 1:** Values assigned to γ and *C* for the LIVE mobile database and the MCL-V database.

Method	γ	*C*
RAVA${}_{SSIM}$ (LIVE mobile database—mobile DMOS)	0.025	pow(2,7)
RAVA${}_{PSNR}$ (LIVE mobile database—mobile DMOS)	1	pow(2,10)
RAVA${}_{SSIM}$ (LIVE mobile database—tablet DMOS)	0.025	pow(2,7)
RAVA${}_{PSNR}$ (LIVE mobile database—tablet DMOS)	0.6	pow(2,10)
RAVA${}_{SSIM}$ (MCL-V database)	3	pow(10,2)
RAVA${}_{PSNR}$ (MCL-V database)	2	pow(2,9)

**Table 2:** Comparison of SCC (Spearman correlation coefficient)—mobile.

Distortion	Co	Wl	Ra	Td	All
PSNR	0.819	0.793	0.598	0.372	0.678
VQM	0.772	0.776	0.648	0.386	0.695
MOVIE	0.774	0.651	0.720	0.158	0.642
MS-SSIM	0.804	0.813	0.738	0.397	0.743
SS-SSIM	0.709	0.725	0.630	0.343	0.650
VIF	0.861	0.874	0.639	0.124	0.744
VSNR	0.874	0.856	0.674	0.317	0.752
NQM	0.850	0.899	0.678	0.238	0.749
**RAVA${}_{\mathrm{PSNR}}$**	0.859	0.873	0.835	**0.554**	0.812
**RAVA${}_{\mathrm{SSIM}}$**	**0.902**	**0.912**	**0.844**	0.507	**0.819**

**Table 3:** Comparison of PCC (Pearson correlation coefficient)—mobile.

Distortion	Co	Wl	Ra	Td	All
PSNR	0.784	0.762	0.536	0.417	0.691
VQM	0.782	0.791	0.591	0.407	0.702
MOVIE	0.810	0.727	0.681	0.244	0.716
MS-SSIM	0.766	0.771	0.709	0.407	0.708
SS-SSIM	0.748	0.731	0.612	0.392	0.664
VIF	0.883	0.898	0.664	0.105	0.787
VSNR	0.849	0.849	0.658	0.427	0.759
NQM	0.832	0.874	0.677	0.365	0.762
**RAVA${}_{\mathrm{PSNR}}$**	0.908	0.905	0.887	**0.659**	0.843
**RAVA${}_{\mathrm{SSIM}}$**	**0.929**	**0.941**	**0.894**	0.603	**0.859**

**Table 4:** Comparison of SCC (Spearman correlation coefficients)—tablet.

Distortion	Co	Wl	Ra	Td	All
PSNR	0.791	0.756	0.446	0.098	0.589
VQM	0.632	0.669	0.436	0.051	0.555
MOVIE	0.774	0.845	**0.771**	0.065	0.679
MS-SSIM	0.660	0.645	0.482	0.140	0.568
SS-SSIM	0.495	0.561	0.368	0.077	0.430
VIF	0.892	0.862	0.671	0.070	0.726
VSNR	0.771	0.705	0.443	0.047	0.593
NQM	0.841	0.808	0.464	0.079	0.661
**RAVA${}_{\mathrm{PSNR}}$**	0.777	0.810	0.613	**0.223**	**0.767**
**RAVA${}_{\mathrm{SSIM}}$**	**0.923**	**0.879**	0.672	−0.084	0.763

**Table 5:** Comparison of PCC (Pearson correlation coefficient)—tablet.

Distortion	Co	Wl	Ra	Td	All
PSNR	0.771	0.732	0.437	0.252	0.635
VQM	0.643	0.735	0.490	0.273	0.735
MOVIE	0.828	0.877	**0.802**	0.071	0.783
MS-SSIM	0.702	0.706	0.564	0.213	0.621
SS-SSIM	0.586	0.590	0.422	0.081	0.489
VIF	0.851	0.854	0.594	0.048	0.764
VSNR	0.775	0.731	0.508	0.220	0.644
NQM	0.812	0.830	0.412	0.120	0.718
**RAVA${}_{\mathrm{PSNR}}$**	0.843	0.869	0.642	**0.279**	**0.848**
**RAVA${}_{\mathrm{SSIM}}$**	**0.957**	**0.921**	0.700	−0.207	0.847

**Table 6:** Comparison of PCC and SCC on the MCL-V database.

	PCC			SCC		
**Distortion**	**Co**	**Scaling**	**All**	**Co**	**Scaling**	**All**
PSNR	0.471	0.463	0.472	0.422	0.493	0.464
SS-SSIM	0.650	0.635	0.650	0.633	0.649	0.648
MS-SSIM	0.617	0.609	0.621	0.609	0.630	0.623
VIF	0.667	0.636	0.660	0.609	0.661	0.655
VADM	0.747	0.728	0.742	0.735	0.741	0.755
FSIM	0.770	0.722	0.750	**0.775**	0.702	0.752
**RAVA${}_{\mathrm{SSIM}}$**	**0.783**	0.709	0.749	0.763	0.696	0.744
**RAVA${}_{\mathrm{PSNR}}$**	0.767	**0.750**	**0.765**	0.750	**0.742**	**0.759**

Note: Co represents distortion type compression.

**Table 7:** Comparison of PCC and SCC on the Netflix Public Dataset.

Methods	PCC	SCC
PSNR	0.536	0.551
SS-SSIM	0.635	0.621
MS-SSIM	0.585	0.631
NQM	0.491	0.579
**RAVA${}_{\mathrm{PSNR}}$**	−0.635	−0.625
**RAVA${}_{\mathrm{SSIM}}$**	**−0.771**	**−0.781**

## Data Availability

No new data were created or analysed in this study. Data sharing is not applicable to this article.

## Abbreviations

RAVA	region-based average video quality assessment
NR-IQA	no-reference image quality assessment
FR	full reference
NIMA	neural image assessment
NR	no-reference
PEVQ	perceptual evaluation of video quality
RR	reduced reference
VQuad-HD	objective perceptual multimedia video quality measurement of HDTV
VQA	video quality assessment
PEXQ	perceptual quality by OPTICOM, a software name
IQA	image quality assessment
ANN	artificial neural network
SSIM	structural similarity
PSD	power spectral density
VSSIM	video structural similarity
PVQA	perceptual video quality assessment
HVS	human visual system
VQA	video quality metric
PSNR	peak signal-to-noise ratio
ANSI	American National Standards Institute
PSNR-HVS	PSNR based on HVS
ITU	International Telecommunication Union
PSNR-HVS-M	PSNR based on between-coefficient contrast masking
VSFA	quality assessment of in-the-wild videos
fps	frames per second
CRF	constant rate factor
PCC	Pearson’s (linear) correlation coefficient
SCC	Spearman’s rank correlation coefficient
